# *Trypanosoma cruzi* P21 recombinant protein modulates *Toxoplasma gondii* infection in different experimental models of the human maternal–fetal interface

**DOI:** 10.3389/fimmu.2023.1243480

**Published:** 2023-10-16

**Authors:** Guilherme de Souza, Samuel Cota Teixeira, Aryani Felixa Fajardo Martínez, Rafaela José Silva, Luana Carvalho Luz, Joed Pires de Lima Júnior, Alessandra Monteiro Rosini, Natália Carine Lima dos Santos, Rafael Martins de Oliveira, Marina Paschoalino, Matheus Carvalho Barbosa, Rosiane Nascimento Alves, Angelica Oliveira Gomes, Claudio Vieira da Silva, Eloisa Amália Vieira Ferro, Bellisa Freitas Barbosa

**Affiliations:** ^1^ Laboratory of Immunophysiology of Reproduction, Institute of Biomedical Sciences, Universidade Federal de Uberlândia, Uberlândia, MG, Brazil; ^2^ Department of Agricultural and Natural Science, Universidade do Estado de Minas Gerais, Ituiutaba, MG, Brazil; ^3^ Institute of Natural and Biological Sciences, Universidade Federal do Triângulo Mineiro, Uberaba, MG, Brazil; ^4^ Laboratory of Trypanosomatids, Institute of Biomedical Sciences, Universidade Federal de Uberlândia, Uberlândia, Brazil

**Keywords:** *Toxoplasma gondii*, congenital toxoplasmosis, *Trypanosoma cruzi*, P21 protein, coinfection, maternal-fetal interface

## Abstract

**Introduction:**

*Toxoplasma gondii* is the etiologic agent of toxoplasmosis, a disease that affects about one-third of the human population. Most infected individuals are asymptomatic, but severe cases can occur such as in congenital transmission, which can be aggravated in individuals infected with other pathogens, such as HIV-positive pregnant women. However, it is unknown whether infection by other pathogens, such as *Trypanosoma cruzi*, the etiologic agent of Chagas disease, as well as one of its proteins, P21, could aggravate *T. gondii* infection.

**Methods:**

In this sense, we aimed to investigate the impact of *T. cruzi* and recombinant P21 (rP21) on *T. gondii* infection in BeWo cells and human placental explants.

**Results:**

Our results showed that *T. cruzi* infection, as well as rP21, increases invasion and decreases intracellular proliferation of *T. gondii* in BeWo cells. The increase in invasion promoted by rP21 is dependent on its binding to CXCR4 and the actin cytoskeleton polymerization, while the decrease in proliferation is due to an arrest in the S/M phase in the parasite cell cycle, as well as interleukin (IL)-6 upregulation and IL-8 downmodulation. On the other hand, in human placental villi, rP21 can either increase or decrease *T. gondii* proliferation, whereas *T. cruzi* infection increases *T. gondii* proliferation. This increase can be explained by the induction of an anti-inflammatory environment through an increase in IL-4 and a decrease in IL-6, IL-8, macrophage migration inhibitory factor (MIF), and tumor necrosis factor (TNF)-α production.

**Discussion:**

In conclusion, in situations of coinfection, the presence of *T. cruzi* may favor the congenital transmission of *T. gondii*, highlighting the importance of neonatal screening for both diseases, as well as the importance of studies with P21 as a future therapeutic target for the treatment of Chagas disease, since it can also favor *T. gondii* infection.

## Introduction

*Toxoplasma gondii* is a protozoan parasite capable of infecting a broad host range, including humans (1, 2). This parasite is the cause of toxoplasmosis, a disease that has an impact on about 30% of the world’s population, with the highest incidences in countries from South America, Africa, and Asia (3, 4).

Most infected individuals are asymptomatic since the host’s immune response can control parasite dissemination (5). The immune response against *T. gondii* is sustained by a pro-inflammatory profile (T helper 1), in which innate and adaptive cells act to control the spread of the parasite in the host organism through effector molecules, such as the cytokines interleukin (IL)-12, interferon (IFN)-γ, tumor necrosis factor (TNF)-α, and macrophage migration inhibitory factor (MIF), which is crucial to parasite growth restriction and the establishment of chronic infection (6–8). However, an exacerbated inflammatory response can lead to tissue damage in the host, being important a counterbalance with anti-inflammatory cytokines, such as IL-4 and IL-10 (9).

Congenital toxoplasmosis is one of the most serious forms of the disease, which is characterized by the cross of *T. gondii* tachyzoites from the mother to the fetus via the placenta during gestation, which can trigger malformations in the offspring, such as hydrocephalus, microcephaly, intrauterine growth restrictions, and blindness (10). Nevertheless, the severity of the disease is influenced by the gestational trimester in which transmission occurs, since the prevalence of congenital transmission rise with the gestational age, as well as the parasite load, the parasite strain, and the maternal immune system (11, 12). The congenital transmission rate ranges from 10-65%, and it is estimated that 1.5 cases occur per 1,000 live births globally, with South America having the highest incidence (4, 13, 14).

Throughout pregnancy, the maternal immune system has to adapt to create a tolerogenic environment for the developing fetus, and at the same time be able to protect the fetus against infection by pathogens (15, 16). In this sense, the gestational period is marked by a change in the profile of pro-inflammatory cytokines (Th1), such as IL-6, IL-8, MIF, and TNF-α, toward an anti-inflammatory profile (Th2), such as IL-4 and IL-10, being important for successful gestation (17). However, these immunological changes that occur during pregnancy, especially in a Th2 environment, may favor susceptibility to infections by pathogens, increasing the risk of vertical transmission through the placenta (18).

In addition, mothers who have been immunocompromised, such as HIV-infected pregnant women, can increase the vertical transmission of *T. gondii* (19–21). Recently, a case of congenital toxoplasmosis was reported in a pregnant woman coinfected with *T. gondii* and severe acute respiratory syndrome coronavirus 2 (SARS-Cov-2) (22). However, it is unknown whether infection by other pathogens, such as *Trypanosoma cruzi*, could influence in *T. gondii* infection at the maternal–fetal interface. Although there are no reports in the literature on humans, coinfection by these two parasites may happen, especially in areas where the incidence of both infections is high, such as South America (23, 24). In Brazil, neonatal screening for congenital toxoplasmosis is recommended for all pregnant women (25). By contrast, testing for Chagas disease during pregnancy is more often recommended to HIV-infected pregnant women or those who have other immunosuppression factors, following the guidelines of the Ministry of Health (26). Thus, the lack of reports about coinfection with *T. gondii* and *T. cruzi* in humans may be related to unreported cases or even a lack of testing, highlighting the importance of prenatal care.

By contrast, natural coinfection by *T. gondii* and *T. cruzi* has already been reported in a female red-necked wallaby that had sudden death but did not present clinical signs but microscopic and molecular analysis confirmed *T. gondii* cysts and *T. cruzi* genotype I (27). In an experimental study, *T. lewisi* infection increased the susceptibility of Sprague–Dawley rats to *T. gondii* infection, and this phenomenon was associated with the reduction in the production of nitric oxide (NO) and inducible nitric oxide synthase (iNOS) in peritoneal macrophages (28), important molecules for the control of *T. gondii* infection (29), favoring its dissemination in the host, showing that in situations of coinfection, the presence of a pathogen can favor infection by another pathogen.

*Trypanosoma cruzi* is a protozoan parasite, being the cause of Chagas disease (30). This disease is considered a major public health problem, affecting about 8 to 10 million people worldwide, with most cases in Latin America where it is endemic in 21 countries, mainly in rural areas where there is greater exposure to the triatomine insect that transmits the parasite (23, 31). Other forms of infection can occur, such as blood transfusion, organ transplantation, laboratory accidents, and consumption of contaminated food and drink, as well as congenital transmission (32). The congenital transmission rate of the parasite is lower compared with *T. gondii* transmission, ranging from 5-10%, and it is estimated that this form of transmission is responsible for about 22.5% of infections in non-endemic countries, although the mechanisms of infection are still poorly known (33–35).

As seen in *T. gondii* infection, the main cytokine involved in *T. cruzi* infection is IFN-γ (36). However, other cytokines such as IL-6, IL-12, and TNF-α contribute to the control of acute infection, but can cause tissue damage in the host in the chronic phase (37). Nevertheless, IL-4 may act to limit this exacerbated response, increasing the susceptibility to the infection (38, 39).

*T. cruzi* releases several molecules for the establishment of infection in the host organism (40). Da Silva et al. (41) discovered one of these molecules, a protein secreted by all evolutive forms of *T. cruzi* and named P21. Studies have shown that this protein not only modulates parasite proliferation (42–44) but also has antiangiogenic and chemotactic activities (45, 46). Additionally, it is responsible for inducing an inflammatory response, being involved in the pathogenesis of Chagas disease, thus configurating a potential therapeutic target for the treatment of the disease (43, 45, 46). These biological activities triggered by P21 are related to its ability to bind to the chemokine CXC motif receptor 4 (CXCR4) of the host cell (47). However, it is unknown whether this protein can also modulate infection by other pathogens, such as *Toxoplasma gondii*, and whether this could lead to aggravation of the infection in the host organism.

In this sense, considering that *T. gondii* and *T. cruzi* can infect and proliferate in the placental environment, that toxoplasmosis and Chagas disease present high rates of infection in endemic regions with potential chances of coinfection, and the absence of reports about coinfection with both parasites typical of unreported or untested cases, we aimed to assess whether *T. cruzi* infection could modulate *T. gondii* infection at the human maternal–fetal interface, and whether P21 could be involved in this modulation in a coinfection scenario. As study models, we used BeWo cells (villous trophoblast) and human placental explants from the third-trimester of pregnancy. These cells and placental explants are widely used as experimental models of the human maternal–fetal interface in the context of *T. gondii* (48–55) and *T. cruzi* infection (56–60).

## Materials and methods

### Cell culture and parasite maintenance

Human villous trophoblast cells (BeWo line) were acquired from the American Type Culture Collection (ATCC, Manassas, VA, USA). The cells were maintained in RPMI 1640 medium (Cultilab, Campinas, SP, Brazil) supplemented with 100 U/mL penicillin, 100 μg/mL streptomycin (Sigma Chemical Co., St Louis, MO, USA), and 10% fetal bovine serum (FBS) (Cultilab) in a humidified incubator at 37°C and 5% CO_2_ (61).

*T. gondii* tachyzoites (RH strain, 2F1 clone) expressing the β-galactosidase gene were kept in BeWo cells in RPMI 1640 medium supplemented with penicillin and streptomycin and 2% FBS at 37°C and 5% CO_2_ (61).

Tissue culture-derived trypomastigotes of *T. cruzi* (TCT, Y strain) were kept in VERO cells in RPMI 1640 medium supplemented with penicillin and streptomycin and 2% FBS at 37°C and 5% CO_2_ (43).

### Human placental explants

Full-term human placentas (36 to 40 weeks of gestation) (n=8) were obtained from pregnant patients after elective cesarean section deliveries at the Clinics Hospital of the Universidade Federal de Uberlândia (HC-UFU), MG, Brazil. Exclusion criteria included pre-eclampsia, hypertension, infectious disease such as toxoplasmosis and Chagas disease, chronic renal disease, cardiac disease, connective tissue disease, diabetes, and other diseases that could hamper the results of this study. After collection, placental tissues were washed in sterile phosphate-buffered saline (PBS) to take out the excess of blood, and the dissection of the villous explants was performed using a stereomicroscope to detach endometrial tissue and fetal membranes up to 1 h after collection. Then, floating terminal chorionic villous containing five to seven free tips per explant were collected, washed with 1x PBS, placed in 96-well plates (one per well), and cultured in 200 µL of RPMI 1640 medium containing 10% FBS, penicillin (100 U/mL), and streptomycin (100 μg/mL) for 24 h at 37°C and 5% CO_2_ (50).

### rP21 purification

Purification of recombinant P21 protein (GenBank: EU004210.1) was carried out as previously described (43, 62).

Briefly, *Escherichia coli* BL21 strain transfected with plasmid pET-28a (+) (Novage) with the gene coding for rP21 were placed in Luria Bertani (LB) medium with the Kanamycin selection antibiotic (50 µg/mL) and kept under agitation overnight at 37°C until the optical density (OD) at 600 nm reached 0.6 to 0.9. Next, 0.5 mM isopropyl β-D-1-thiogalactopyranoside (IPTG, Sigma) was added to induce expression of rP21. For bacterial lysis, lysozyme (50 mg/mL) was added for 20 min, and then the Sonicador Branson Sonifier 450 was used for 20 min (1-min cycle with 30-sec interval). The bacterial lysate pellet was resuspended in 9 M urea buffer solution and incubated with a nickel resin column in agitation for 2 h at 4°C. rP21 has a histidine tail with an affinity for nickel resin, sticking to the column. Then, the column was washed to capture rP21, as follows (1): three washes in binding buffer (5 mM imidazole, 500 mM NaCl, Tris-HCl 20 mM pH 8.0, 6 M urea) (2); three washes in wash buffer (20 mM imidazole, 500 mM NaCl, 20 mM Tris-HCl pH 8.0, 6 M urea); and (3) four elutions with elution buffer (1 M imidazole, 50 mM NaCl, 20 mM Tris-HCl pH 8.0, 6 M urea). The buffers used had increasing concentrations of imidazole (5 mM, 20 mM, and 1 M, respectively) as it has a greater affinity for the nickel resin than the histidine tail present in the rP21, thus allowing the release of rP21 in the supernatant. Dialysis was performed in 1x PBS for 48 h under agitation at 4 °C with a 3.5 kDa micropore dialysis membrane (Spectra/131198).

After purification, the protein was incubated with a Polymyxin B column (Sigma, P1411) to remove bacterial endotoxins and the concentration was quantified with the Bradford method (63). rP21 purity was analyzed with 13% SDS-PAGE gel with Coomassie blue staining.

### Cell viability assay

The viability of BeWo cells treated with rP21 was evaluated by MTT assay [(3-(4,5-dimethylthiazol-2-yl)-2,5-diphenyltertrazolin bromide)], as described by Mosmann (64).

BeWo cells (3×10^4^/200 μL) were seeded in 96-well plates in 10% FBS medium for 24 h at 37 °C and 5% CO_2_. Then, the cells were treated with rP21 (10, 20, 40, or 80 µg/mL) in medium for additional 24 h. The rP21 concentrations used were based on previous studies (42, 43, 46, 47, 65). As control, cells were treated only with 10% FBS medium.

Afterwards, the supernatants were collected and stored at -80 °C for further cytokine measurement, and the cells were incubated with MTT (5 mg/mL) diluted in 10% FBS medium for 4 h at 37°C and 5% CO_2_. Then, the formazan crystals were solubilized in 10% sodium dodecyl sulfate (SDS, Sigma) and 50% N, N- dimethyl formamide (Sigma). The absorbance was measured at 570 nm in a microplate spectrophotometer (VersaMax ELISA Microplate Reader, Molecular Devices, Sunnyvale, CA, USA). The viability of BeWo cells was expressed as percentage of viable cells (cell viability %) by comparing the treatments with the cells treated only with 10% FBS medium (medium, 100% viability). Two independent experiments with eight replicates were performed.

### *T. gondii* invasion and intracellular proliferation assay in BeWo cells treated with rP21 and/or infected with *T. cruzi*

The impact of *T. cruzi* infection (TCT) and/or rP21 treatment on *T. gondii* invasion (3 h of infection) and intracellular proliferation (24 h of infection) in BeWo cells were evaluated by means of the β-galactosidase assay (50).

**Figure 1:**
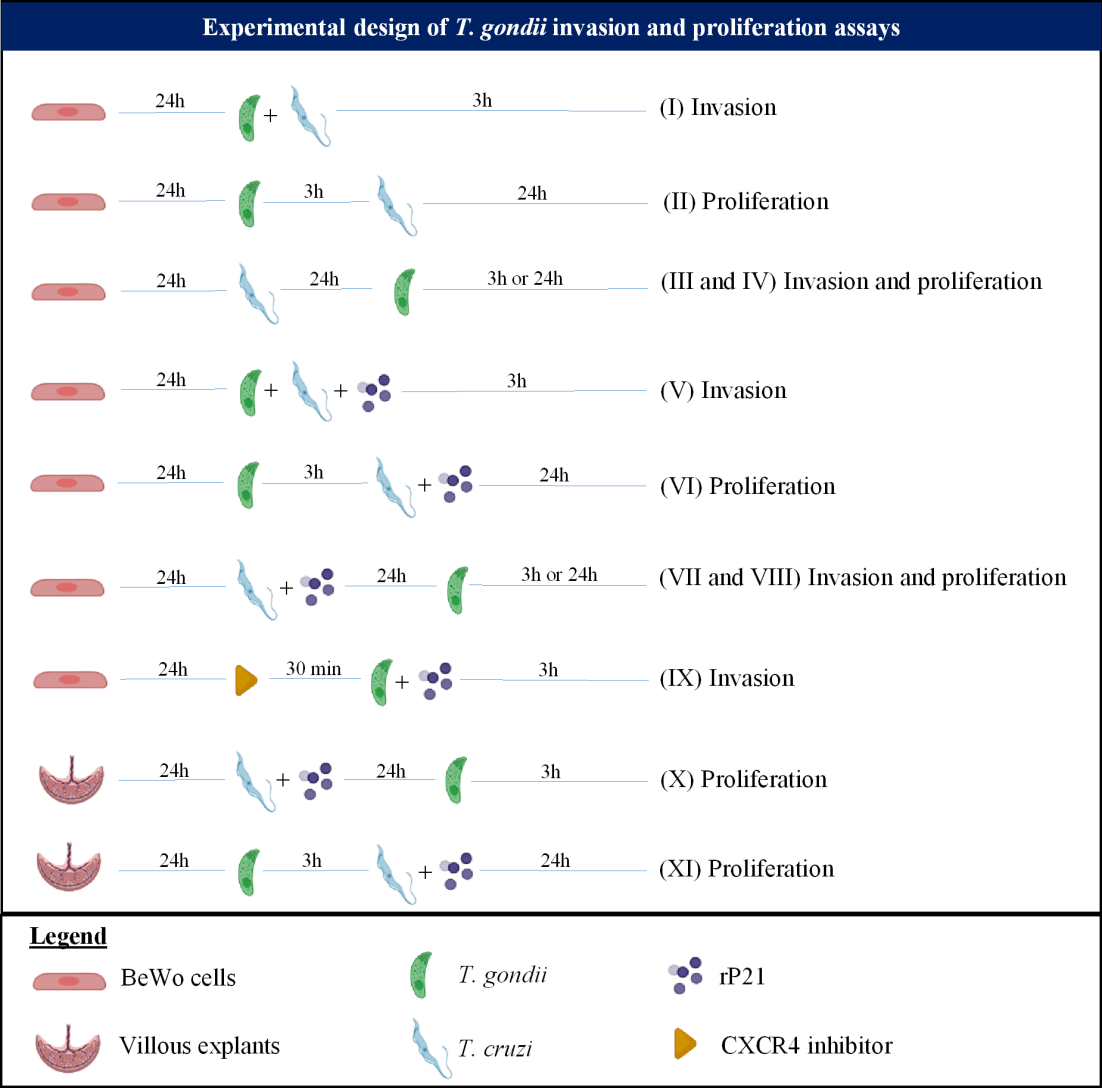
Experimental design summarizing the *T. gondii* invasion and proliferation experiments carried out in this study.

For all *T. gondii* invasion and proliferation experiments, BeWo cells (3×10^4^/200 μL) were seeded in 96-well plates in 10% FBS medium for 24 h at 37 °C and 5% CO_2_. Also, in order to remove non-internalized parasites after the period of infection stated below, the cells were washed thrice with 1x PBS. Nine different experimental protocols were carried out, as represented in **Figure 1**:

*(I) Simultaneous infection with T. gondii and T. cruzi*: BeWo cells were infected with *T. gondii* tachyzoites with multiplicity of infection (MOI) of 3:1, and *T. cruzi* trypomastigotes (1:1) simultaneously for 3 h in medium. As control, cells were infected only with *T. gondii* (infected medium). After 3 h, the cells were washed and then submitted to the β-galactosidase assay for analysis of *T. gondii* invasion.
*(III and IV) Pre-infection with T. cruzi*: BeWo cells were pre-infected with *T. cruzi* (1:1) in medium for 24 h. Next, the cells were washed and then were infected with *T. gondii* (3:1) for 3 h in medium. As control, cells were infected only with *T. gondii* (infected medium). Then, the cells were washed, and two experimental conditions were evaluated: (III) the cells were submitted to the *T. gondii* invasion assay; or (IV) the cells were treated with medium for additional 24 h for analysis of *T. gondii* proliferation, both by means of the β-galactosidase assay.
*(V) Simultaneous infection and/or treatment with rP21:* BeWo cells were infected with *T. gondii* tachyzoites (3:1) and *T. cruzi* trypomastigotes (1:1) simultaneously in the presence or absence of rP21 (40 μg/mL) for 3 h in medium. In parallel, cells were infected only with *T. gondii* and simultaneously treated with rP21 (40 μg/mL). As control, cells were infected only with *T. gondii* (infected medium). Afterwards, the cells were washed, and two experimental conditions were evaluated: (V) the cells were submitted to the *T. gondii* invasion assay or the cells were treated with medium for additional 24 h for analysis of *T. gondii* proliferation, both by means of the β-galactosidase assay.
*(VI) Pre-infection with T. gondii and treatment with rP21*: BeWo cells were infected with *T. gondii* tachyzoites (3:1) for 3 h in medium. Next, the cells were washed and then were treated with rP21 (40 µg/ml), or infected with *T. cruzi* trypomastigotes (1:1), or infected with *T. cruzi* and simultaneously treated with rP21 in medium for an additional 24 h. As control, cells were infected only with *T. gondii* (infected medium). Afterwards, the supernatant was collected and stored at -80 °C for further cytokine measurement, and the cells were submitted to the β-galactosidase assay for analysis of *T. gondii* proliferation.
*(VII and VIII) Pre-infection with T. cruzi and treatment with rP21*: BeWo cells were pre-treated with rP21 (40 µg/mL), or pre-infected with *T. cruzi* (1:1), or pre-infected with *T. cruzi* and simultaneously treated with rP21 in medium for 24 h. Next, the treatment and the parasites were removed by washing with 1x PBS, and the cells were infected with *T. gondii* (3:1) for 3 h in medium. As control, cells were infected only with *T. gondii* (infected medium). Afterwards, the cells were washed, and two experimental conditions were evaluated: (VII) the cells were submitted to the *T. gondii* invasion assay; or (VIII) the cells were treated with medium for an additional 24 h for analysis of *T. gondii* proliferation, both by means of the β-galactosidase assay.
*(IX) CXCR4 blockade*: BeWo cells were pre-treated or not with the CXCR4 inhibitor AMD3100 (30 µM, Merck, Darmstadt, Germany) for 30 min, as previously described (47). Next, the inhibitor was removed and the cells were infected with *T. gondii* tachyzoites (3:1) and treated or not with rP21 (40 μg/mL) for 3 h in medium. As control, cells were only infected (infected medium). Afterwards, the cells were washed and then submitted to the β-galactosidase assay for analysis of *T. gondii* invasion.

*T. gondii* invasion and intracellular proliferation was obtained according to a reference curve with free tachyzoites (from 1 × 10^6^ to 15.6 × 10^3^). For all of the above experiments, data are presented as percentage of *T. gondii* invasion or proliferation in comparison with the cells only infected with *T. gondii* (medium, 100% invasion or proliferation). Three independent experiments with eight replicates were performed.

### Analysis of actin cytoskeletal polymerization in BeWo cells infected with *T. gondii* and treated with rP21

The analysis of the actin cytoskeleton polymerization was evaluated by confocal fluorescence microscopy.

BeWo cells (1×10^5^/500 μL) were seeded in 24-well plates on glass coverslips (13 mm) in 10% FBS medium for 24 h at 37°C and 5% CO_2_. Next, the cells were infected or not with *T. gondii* tachyzoites (3:1) and treated or not simultaneously with rP21 (40 μg/mL) for 3 h. As controls, cells were uninfected and untreated (uninfected medium) or infected and untreated (infected medium). Afterwards, the cells were washed thrice with 1x PBS to remove non-internalized parasites and then fixed with paraformaldehyde (4%) for 30 min. Next, the coverslips were washed thrice with 1x PBS and incubated overnight with a mouse monoclonal primary antibody anti-*T. gondii* (SAG1/p30) (Abcam TP3 #ab8313, Cambridge, UK) [diluted 1:500 in PBS solution containing 0.15% gelatin, 0.1% sodium azide, and 1% saponin (PGN-saponin)]. Then, the coverslips were washed thrice with 1x PBS and incubated with Alexa Fluor 488-conjugated anti-mouse IgG (Invitrogen #A11001, Waltham, MA, USA) (diluted 1:500 in PGN-0.01% saponin solution), sodium isothiocyanate tetramethylrhodamine (TRITC) conjugated with phalloidin (Sigma, P1951) (diluted 1:50 in PGN-saponin 0.01%), and TO-PRO-3 (Life Technologies, Carlsbad, CA, USA) (diluted 1:450 in PGN-saponin 0.01%) for 1 h at room temperature under darkness to label *T. gondii* tachyzoites, actin, and nuclei, respectively. Afterwards, the coverslips were washed thrice with 1x PBS and mounted on glass slides and analyzed in a confocal fluorescence microscopy (Zeiss, LSM 510 Meta, Germany) with an inverted microscope (Zeiss Axiovert 200 M). The mean actin fluorescence intensity was evaluated in 100 cells in triplicate of each condition on ImageJ software (National Institutes of Health, USA).

### Analysis of the impact of rP21 on *T. gondii* cell cycle

The analysis of the cell cycle of *T. gondii* was evaluated using flow cytometry, as previously described (66).

BeWo cells (1×10^5^/500 μl) were seeded in 24-well plates in 10% FBS medium for 24 h at 37°C and 5% CO_2_. Next, the cells were infected with *T. gondii* tachyzoites (3:1) for 3 h. Then, the cells were washed three times with 1x PBS to remove non-internalized parasites, and the cells were treated with rP21 (40 µg/ml) in medium for an additional 24 h. As control, cells were infected and untreated (infected medium). Afterwards, the cells were washed thrice with 1x PBS to remove extracellular parasites, and a cell scraper was used to detach the cells. The intracellular parasites were isolated through a syringe with 21- and 26-gauge needles. The parasite/cell lysate was centrifuged at 1,500 rpm for 30 sec to remove cell debris, and the supernatant was centrifuged at 1,500 rpm for 5 min to pellet the isolated parasites. The parasites from each condition were washed with 1x PBS and fixed with 70% ethanol at 4°C overnight. Next, fixed parasites were washed with 1x PBS and incubated overnight with a mouse monoclonal primary antibody anti-*T. gondii* (SAG1/p30) (Abcam TP3 #ab8313) (diluted 1:500 in PGN-saponin 0.01%). Then, the parasites were washed with 1x PBS and incubated with Alexa Fluor 488-conjugated anti-mouse IgG (Invitrogen #A11001), 10 μg/mL of propidium iodide, and 100 μg/mL of RNAse A (all reagents diluted in 1x PBS) for 45 minutes at room temperature under darkness. The DNA content for parasite cell cycle analysis was performed using the Cytoflex flow cytometer (Beckman Coulter, USA), and data collected using FlowJo software (version 7.6.3). Two independent experiments with five replicates were performed.

### *T. gondii* reinfection assay

To assess whether treatment with rP21 could be compromising the infective capacity of *T. gondii*, we performed a reinfection assay to evaluate the parasite invasion.

BeWo cells (1×10^6^/2000 μL) were seeded in 6-well plates in 10% FBS medium for 24 h at 37°C and 5% CO_2_. Next, the cells were infected with *T. gondii* (3:1) for 3 h in medium. Then, the cells were washed thrice with 1x PBS to remove non-internalized parasites, and the cells were treated with rP21 (40 µg/mL) in medium for an additional 24 h. Afterwards, the cells were washed three times with 1x PBS to remove extracellular parasites, and the intracellular parasites were isolated using syringes as described above. Parasites isolated from each condition were then used to reinfect new fresh BeWo cells previously seeded in 96-well plates (3×10^4^/200 μL) (3:1) in medium for 3 h. As control, cells were infected with parasites from cells that were not treated with rP21 (infected medium). Next, the cells were washed thrice with 1x PBS to remove non-internalized parasites and then submitted to the β-galactosidase assay for analysis of *T. gondii* invasion. Three independent experiments with eight replicates were performed.

### Viability of human placental explants treated with rP21

The viability of human placental explants treated with rP21 was evaluated by MTT assay, as previously described (52, 66).

Human placental explants were collected as previously described and cultured in 96-well plates in 10% FBS medium for 24 h at 37°C and 5% CO_2_. Next, the villous explants were treated with rP21 (40 μg/mL) in medium for an additional 24 h. As control, the villous explants were treated only with 10% FBS medium (medium).

Afterwards, the supernatants were collected and stored at -80°C for further cytokine measurement, and the villous explants were submitted to the MTT assay, as described above. The viability of villi was expressed as percentage viability of villous explants in relation to explants treated only with medium (medium, 100% viability).

Additionally, the villi treated or not with rP21 were collected for morphological analysis using hematoxylin and eosin staining (50).

### *T. gondii* intracellular proliferation in human placental explants treated with rP21 and/or infected with *T. cruzi*

The *T. gondii* intracellular proliferation in human placental explants treated with rP21 and/or infected with *T. cruzi* was evaluated by the β-galactosidase assay (50).

The villous explants were cultured in 96-well plates for 24 h in 10% FBS medium at 37°C and 5% CO_2_. Then, two different experimental protocols were carried out, as demonstrated in **Figure 1**:

*(X) Pre-treatment and/or pre-infection with T. cruzi:* The villous explants were pre-treated with rP21 (40 µg/mL), or pre-infected with *T. cruzi* trypomastigotes (1x10^5^), or pre-infected with *T. cruzi* and treated simultaneously with rP21 in medium for 24 h. Next, the treatment and parasites were removed by washing with 1x PBS, and the villi were infected with *T. gondii* (1x10^6^) for an additional 24 h in medium. As control, the villous explants were only infected with *T. gondii* (infected medium). Afterwards, the villi were collected for analysis of *T. gondii* intracellular proliferation by the β-galactosidase assay. Three independent experiments with eight replicates were performed.
*(XI) Pre-infection with T. gondii:* The villous explants were pre-infected with *T. gondii* tachyzoites (1x10^6^) for 24 h in medium. Next, the parasites were removed by washing with 1x PBS, and the villi were treated with rP21 (40 µg/mL), or infected with *T. cruzi* trypomastigotes (1x10^5^), or infected with *T. cruzi* and simultaneously treated with rP21 in medium for an additional 24 h. As control, the villous explants were only infected with *T. gondii* (infected medium). Afterwards, the supernatant was collected and stored at -80 °C for further cytokine measurement, and the villi were collected for analysis of *T. gondii* intracellular proliferation by the β-galactosidase assay. Three independent experiments with eight replicates were performed.

The *T. gondii* intracellular proliferation in villous explants samples was performed by adding 150 µL radioimmunoprecipitation assay buffer (RIPA) (50 mM Tris-HCl, 150 mM NaCl, 1% Triton X-100, 1% sodium deoxycholate, and 0.1% sodium dodecyl sulfate, pH 7.5) supplemented with a protease inhibitor cocktail (Complete, Roche Diagnostic, Mannheim, Germany) to each villi and homogenizing the samples on ice for protein extraction. The lysate was centrifuged at 21,000 × g for 15 min at 4°C and the supernatant was collected to measure the protein total (µg/mL) by Bradford assay (63). Aliquots of 20 µL of each sample were used to determine *T. gondii* intracellular proliferation by the β-galactosidase assay, as described above. The number of tachyzoites were normalized according to the protein concentration of each villi, showing the number of tachyzoites per µg of tissue. The data were expressed as percentage (%) of *T. gondii* proliferation: the mean number of tachyzoites from control (untreated and infected villi) corresponded to 100% of the parasite proliferation, and the number of tachyzoites from each condition was transformed into a percentage according to 100% of the parasite proliferation from the control.

### Cytokine measurement

The human cytokines IL-4, IL-6, IL-8, IL-10, MIF, and TNF-α were measured in BeWo cell supernatants and human placental explants. The measurement of these cytokines in the supernatants was performed using a double-antibody sandwich enzyme-linked immunosorbent assay (ELISA), according to the manufacturers’ instructions (BD Biosciences, San Jose, CA, USA or R&D Systems, Minneapolis, MN, USA). Data were expressed in pg/mL according to a standard curve of each cytokine for BeWo cells, while for villous explants data were normalized according to the protein concentration of each villi, where data about cytokines were obtained by the ratio between the concentration of cytokines in pg/mL and the concentration of total protein from the Bradford assay in µg/mL, resulting in pg/µg of tissue (67).

The detection limits of each cytokine were: IL-4, IL-10, and TNF-α (7.8 pg/mL), IL-6 (4.7 pg/mL), IL-8 (3.12 pg/mL), and MIF (31.2 pg/mL).

### Ethical approval

The present research protocol using human tissue samples was performed in accordance with relevant guidelines and regulations, being approved by the Ethics Committee of the Universidade Federal de Uberlândia, MG, Brazil, with approval number 3.679.426. A consent term was obtained from all participants included in this study.

## Results

### *T. cruzi* infection increases invasion and decreases proliferation of *T. gondii* in BeWo cells

**Figure 2:**
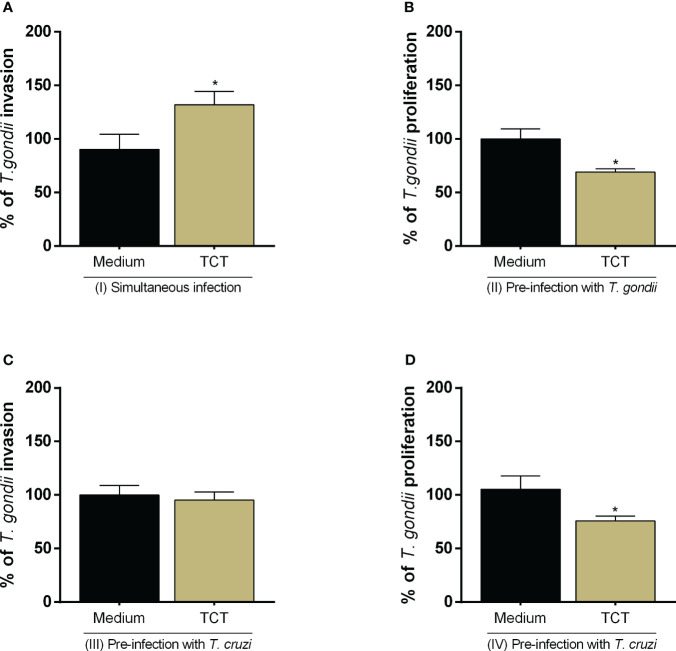
*T. gondii* invasion and proliferation in BeWo cells infected with *T. cruzi*. BeWo cells were simultaneously infected with *T. gondii* and *T. cruzi* **(A)**, or infected with *T. gondii* and subsequently with *T. cruzi* **(B)**, or pre-infected with *T. cruzi* and later with *T. gondii* **(C, D)**. *T. gondii* invasion **(A, C)** and intracellular proliferation **(B, D)** were analyzed by the β-galactosidase assay and are presented as percentage (%) of *T. gondii* invasion or proliferation in relation to cells only infected with *T. gondii* (infected medium). Data are shown as means ± SEM of three independent experiments with eight replicates. Differences between groups were analyzed with one-way ANOVA test with Bonferroni’s multiple comparisons post-test. Significant differences in relation to cells only infected with *T. gondii* (^*^).

We assessed whether *T. cruzi* infection would modulate *T. gondii* invasion and proliferation in the host cell (**Figures 2A–D**). We observed that simultaneous infection with both parasites increased *T. gondii* invasion (^*^ *P* = 0.0465) in relation to cells only infected with *T. gondii* (infected medium) (**Figure 2A**), but the pre-infection with *T. cruzi* did not show the same effect (**Figure 2C**). In addition, we observed that pre- and post-*T. cruzi* infection decreased *T. gondii* proliferation (^*^ *P* = 0.0353 and ^*^ *P* = 0.0111, respectively) in relation to cells only infected with *T. gondii* (infected medium) (**Figures 2B, D**).

### rP21 does not alter viability, increases invasion, and decreases proliferation of *T. gondii* in BeWo cells

**Figure 3:**
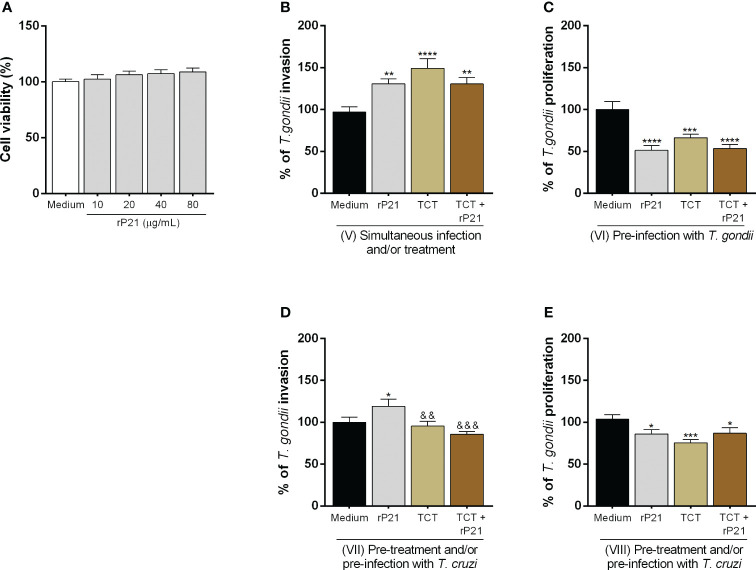
Analysis of the role of rP21 in the viability, *T. gondii* invasion, and proliferation in BeWo cells. BeWo cells were treated with rP21 for 24 h and submitted to the MTT assay for cell viability analysis **(A)** and are presented as percentage (%) of cell viability in relation to untreated cells (untreated medium). Data are shown as means ± SEM of two independent experiments with eight replicates. In addition, BeWo cells were infected with *T. gondii* and treated with rP21 and/or infected with *T. cruzi* simultaneously **(B)**, or infected with *T. gondii* and subsequently treated and/or infected with *T. cruzi* **(C)**, or pre-treated and/or pre-infected with *T. cruzi* and subsequently with *T. gondii* **(D, E)**. *T. gondii* invasion **(B, D)** and intracellular proliferation **(C, E)** were analyzed with the β-galactosidase assay and are presented as percentage (%) of *T. gondii* invasion or proliferation in relation to cells only infected with *T. gondii* (infected medium). Data are shown as means ± SEM of three independent experiments with eight replicates. Differences between groups were analyzed by one-way ANOVA test with Bonferroni’s multiple comparisons post-test. Significant differences in relation to cells only infected with *T. gondii* (^*^) or infected and treated with rP21 (^&^).

As described above, previous studies showed that P21 protein of *T. cruzi* is able to modulate the parasite proliferation itself (42–44). Thus, we evaluated whether the effect promoted by *T. cruzi* infection on *T. gondii* infection could be related to its protein P21. We observed that this protein did not alter cell viability at any concentration in relation to untreated cells (uninfected medium) (**Figure 3A**). For further experiments, we chose only the concentration of 40 μg/mL.

Next, we assessed whether treatment with rP21 could modulate both *T. gondii* invasion and proliferation (**Figures 3B–E**). We observed that simultaneous treatment with rP21 plus *T. gondii* infection (^**^ *P* = 0.0034), simultaneous infection with *T. cruzi* and *T. gondii* (^****^ *P* < 0.0001), and simultaneous infection with *T. cruzi* and *T. gondii* plus rP21 (^**^ *P* = 0.006) increased *T. gondii* invasion in relation to cells only infected with *T. gondii* (infected medium) (**Figure 3B**). However, the simultaneous treatment with rP21 (^**^ *P* = 0.001), simultaneous infection with *T. cruzi* (^*^ *P* = 0.0215), and simultaneous infection with *T. cruzi* plus rP21 (^****^ *P* < 0.0001) decreased *T. gondii* proliferation in relation to cells only infected with *T. gondii* (infected medium) (**Supplementary Figure 1**). In addition, pre-infection with *T. gondii* and subsequent treatment with rP21 and/or infection with *T. cruzi* decreased *T. gondii* proliferation in relation to cells only infected with *T. gondii* (infected medium) (rP21: ^****^ *P* < 0.0001; TCT: ^***^ *P* = 0.0005; TCT + rP21: ^****^ *P* < 0.0001) (**Figure 3C**).

Furthermore, we observed that pre-treatment with rP21 (^*^ *P* = 0.0350) increased *T. gondii* invasion in relation to cells only infected with *T. gondii* (infected medium) (**Figure 3D**). On the other hand, the pre-infection by *T. cruzi* in the absence (^&&^ *P* = 0.0094) or presence of rP21 (^&&&^ *P* = 0.0005) decreased *T. gondii* invasion in relation to cells infected with *T. gondii* and treated with rP21, but no change was observed in comparison with cells only infected with *T. gondii* (infected medium) (**Figure 3D**). In addition, pre-treatment with rP21 (^*^ *P* = 0.0182), or pre-infection with *T. cruzi* (^***^ *P* = 0.0002) or pre-infection and simultaneous treatment with rP21 (^*^ *P* = 0.0311) decreased *T. gondii* proliferation in relation to cells only infected with *T. gondii* (infected medium) (**Figure 3E**).

### The greater invasion of *T. gondii* promoted by rP21 is dependent on its binding to CXCR4 and the actin cytoskeleton polymerization

**Figure 4:**
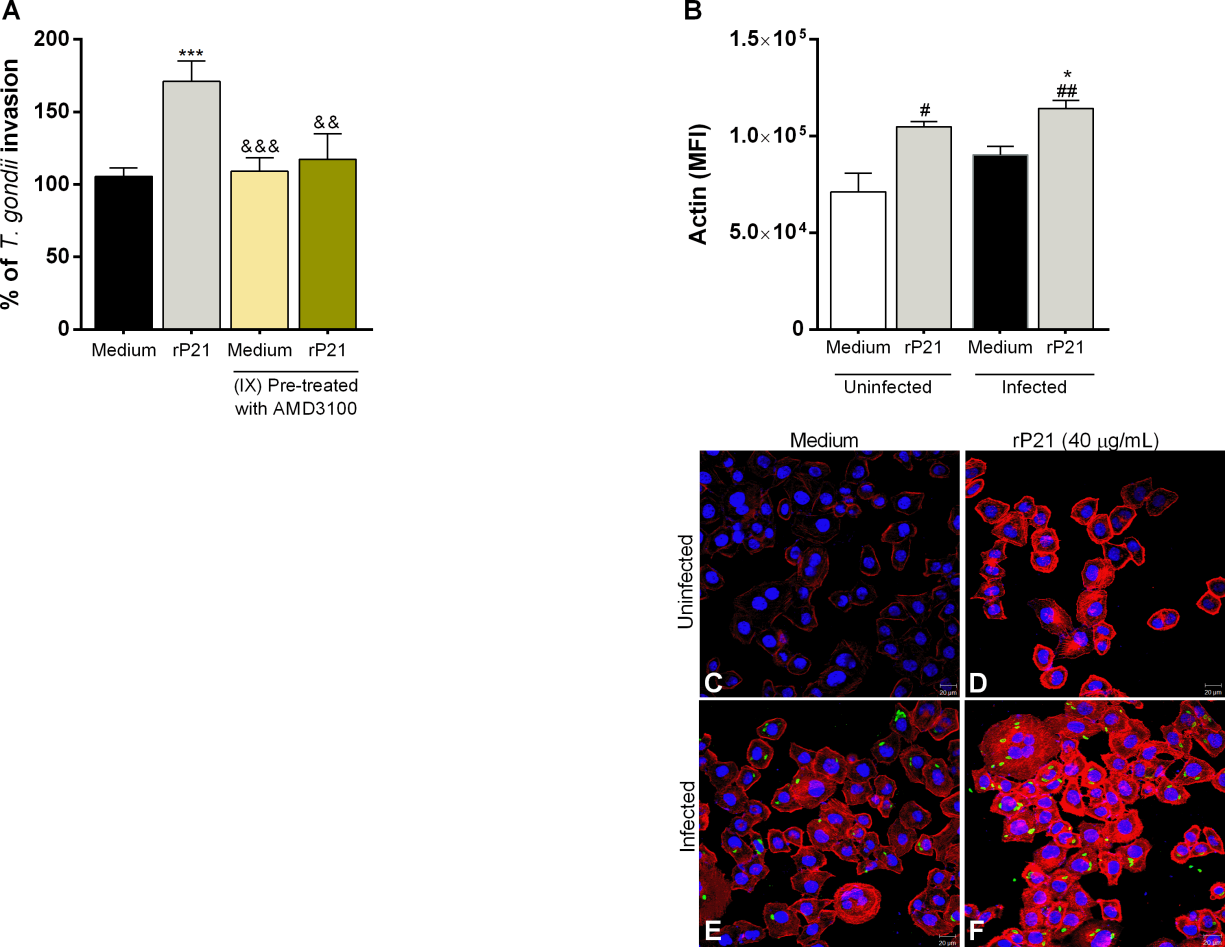
Analysis of the role of CXCR4 and actin cytoskeleton of BeWo cells in *T. gondii* invasion. BeWo cells were pre-treated or not with AMD3100 for 30 min and subsequently infected with *T. gondii* and treated simultaneously with rP21 for 3 h. *T. gondii* invasion **(A)** was analyzed with the β-galactosidase assay and are presented as percentage (%) of *T. gondii* invasion in relation to cells only infected with *T. gondii* (infected medium). Data are shown as means ± SEM of three independent experiments with eight replicates. Differences between groups were analyzed with one-way ANOVA test with Bonferroni’s multiple comparisons post-test. Significant differences in relation to cells only infected with *T. gondii* (^*^) or infected and treated with rP21 (^&^). In addition, BeWo cells were seeded on glass coverslips and infected with *T. gondii* and treated with rP21 simultaneously for 3 h. Then, the cells were fixed and labeled for the actin cytoskeleton (red), *T. gondii* (green), and nuclei (blue). The actin cytoskeleton was analyzed under confocal fluorescence microscopy **(B)**. The mean actin fluorescence intensity was evaluated in 100 cells in triplicate of each condition in ImageJ software (National Institutes of Health, USA). Differences between groups were analyzed by Kruskall–Wallis test with Dunn’s multiple comparison post-test. Representative images are shown of uninfected and untreated BeWo cells **(C)**, uninfected and treated with rP21 **(D)**, infected and untreated **(E)**, and infected and treated with rP21 **(F)**. Significant differences in relation to uninfected and untreated cells (^#^) or infected and untreated (^*^). Scale bar: 20 µm.

We evaluated the role of CXCR4 and the actin cytoskeleton in *T. gondii* invasion in BeWo cells treated with rP21 (**Figures 4A–F**). It is important to highlight that in this set of experiments, the *T. gondii* infection was simultaneous with the rP21 treatment and without the presence of *T. cruzi*.

We observed that, as seen previously, rP21 increased *T. gondii* invasion (^***^ *P* = 0.0007) in relation to cells only infected (infected medium). Interestingly, BeWo cells infected and treated with the CXCR4 inhibitor AMD3100 (^&&&^ *P* = 0.0008) or infected and treated with AMD3100 plus rP21 (^&&^ *P* = 0.0041) reduced the parasite invasion in comparison with cells only treated with rP21 (**Figure 4A**).

As for actin cytoskeleton polymerization (**Figure 4B**), we observed that rP21, regardless of *T. gondii* infection, increased actin cytoskeleton polymerization in relation to uninfected and untreated cells (^#^ *P* = 0.0416; ^##^ *P* = 0.0047) (uninfected medium). In addition, cells infected and treated with rP21 increased actin cytoskeletal polymerization in relation to cells only infected (^*^ *P* = 0.0416) (infected medium).

Representative images (**Figures 4C–F**) are shown of uninfected and untreated BeWo cells (4C), uninfected and treated with rP21 (4D), infected and untreated (4E), and infected and treated with rP21 (4F), where one can observe that in the presence of rP21 there is a greater actin cytoskeleton polymerization of the host cell (red), as well as a greater amount of *T. gondii* inside the cell (green).

### rP21 causes an arrest in the S/M phase in *T. gondii* cell cycle, but does not compromise its capacity to infect new host cells

**Figure 5:**
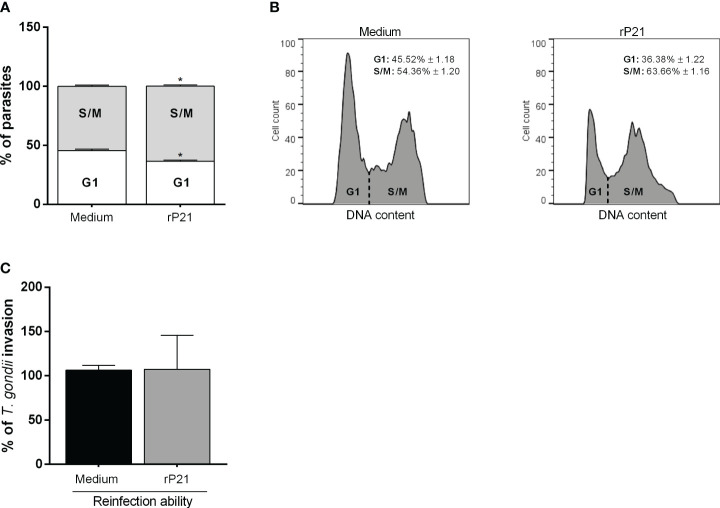
Cell cycle analysis and reinfection capacity of *T. gondii*. BeWo cells were infected with *T. gondii* for 3 h and then treated with rP21 for 24 h. The intracellular parasites were isolated through syringes with 21- and 26-gauge needles, and submitted to cell cycle analysis **(A, B)** or reinfection assay **(C)**. Cell cycle data are shown as percentage (%) of parasites in G1 and S/M phase, and are presented as means ± SEM of one experiment with five replicates. For the reinfection assay, the data are presented as percentage (%) of *T. gondii* invasion in relation to the parasites untreated (infected medium). Data are shown as means ± SEM of three independent experiments with eight replicates. Differences between groups were analyzed with one-way ANOVA test with Bonferroni’s multiple comparisons post-test. Significant differences in relation to parasites untreated (^*^).

We assessed whether the decrease in *T. gondii* proliferation triggered by rP21 could be involved with the progression in the parasite cell cycle. We observed that treatment with rP21 after *T. gondii* infection decreased the number of parasites in the G1 phase (^*^ *P* = 0.0145) and increased the number of parasites in the S/M phase (^*^ *P* = 0.0136) in relation to the parasites that were not treated (infected medium) (**Figures 5A, B**).

In addition, we observed that parasites that were treated for 24 h with rP21 and allowed to reinfect fresh new cells showed similar invasive capacity if compared with parasites that were not treated (infected medium) (**Figure 5C**).

### rP21 induces an increase in IL-6 and decreases IL-8 production

**Figure 6:**
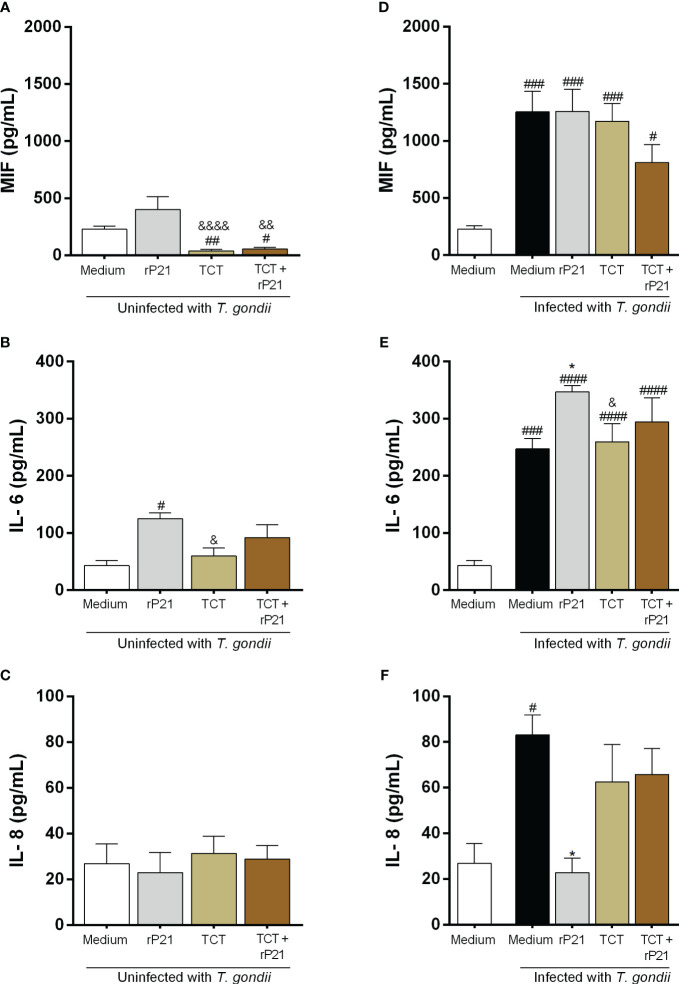
Cytokine production in BeWo cells. The supernatants of BeWo cells infected or not with *T. gondii* and/or *T. cruzi* and treated or not with rP21 were submitted to ELISA assay for measurement of MIF **(A, D)**, IL-6 **(B, E)**, and IL-8 **(C, F)**. Data are presented in pg/mL according to the standard curve for each cytokine, and are shown as means ± SEM of three independent experiments with three replicates. Differences between groups were analyzed by means of one-way ANOVA test with Bonferroni’s multiple comparisons post-test or by means of Kruskall–Wallis test with Dunn’s multiple comparisons post-test. Significant differences in relation to uninfected and untreated cells (^#^) or infected or not with *T. gondii* and treated with rP21 (^&^) or only infected with *T. gondii* (^*^).

Next, since rP21 and *T. cruzi* decrease *T. gondii* proliferation, we evaluated whether this protein, as well as *T. cruzi*, would be interfering in the host immune response through cytokine production in BeWo cells (**Figures 6A–F**).

We observed that cells only infected with *T. cruzi*, in the absence or presence of rP21, decreased MIF production in relation to uninfected and untreated cells (uninfected medium) (TCT: ^##^ *P* = 0.0029; TCT + rP21: ^#^ *P* = 0.0143), as well as in relation to uninfected cells and treated with rP21 (TCT: ^&&&&^ *P* = 0.0008; TCT + rP21: ^&&^ *P* = 0.0049) (**Figure 6A**). On the contrary, cells only infected with *T. gondii*, or infected and treated with rP21, or infected with *T. gondii*/*T. cruzi* in the absence or presence of rP21 increased MIF production in relation to uninfected and untreated cells (uninfected medium) (Infected medium: ^###^ *P* = 0.0003; rP21: ^###^ *P* = 0.0002; TCT: ^###^ *P* = 0.0007; TCT + rP21: ^#^ *P* = 0.0277) (**Figure 6D**).

Regarding IL-6 production, we observed that cells only treated with rP21 increased the production of this cytokine in relation to uninfected and untreated cells (^#^ *P* = 0.0117) (uninfected medium) (**Figure 6B**). Conversely, cells infected only with *T. cruzi* had a lower IL-6 production in relation to cells only treated with rP21 (^&^ *P* = 0.0424) (**Figure 6B**). In addition, we observed that cells only infected with *T. gondii*, or infected and treated with rP21, or infected with *T. gondii*/*T. cruzi* in the absence or presence of rP21 increased IL-6 production in relation to uninfected and untreated cells (uninfected medium) (infected medium: ^###^ *P* = 0.0001; rP21: ^####^ *P* < 0.0001; TCT: ^####^ *P* < 0.0001; TCT + rP21: ^####^ *P* < 0.0001) (**Figure 6E**). Furthermore, we observed that cells only infected with *T. gondii* and treated with rP21 increased IL-6 in relation to cells only infected with *T. gondii* (^*^ *P* = 0.0269), while infection with *T. gondii*/*T. cruzi* decreased IL-6 production in relation to cells infected with *T. gondii* and treated with rP21 (^&^ *P* = 0.0497) (**Figure 6E**).

Concerning IL-8 production, no changes were detected in the absence of *T. gondii* (**Figure 6C**). However, we observed that cells only infected with *T. gondii* increased the production of this cytokine in relation to uninfected and untreated cells (^#^ *P* = 0.039) (**Figure 6F**), while treatment with rP21 decreased IL-8 production in relation to cells only infected with *T. gondii* (infected medium) (^*^ *P* = 0.0218) (**Figure 6F**).

IL-4, IL-10, and TNF-α showed values below detection levels in all conditions tested (data not shown).

### rP21 and the presence of *T. cruzi* increased *T. gondii* proliferation in human placental explants

**Figure 7:**
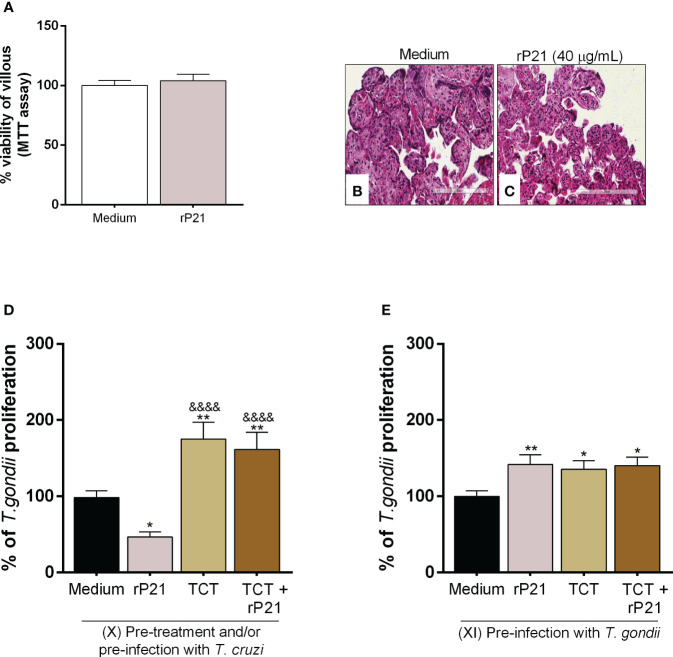
Analysis of rP21 treatment and *T. cruzi* infection in *T. gondii* proliferation in human placental explants. Human placental explants were treated with rP21 for 24 h and submitted to the MTT assay **(A)**. Data are presented as percentage (%) viability of villous in relation to untreated villous (untreated medium). Data are shown as means ± SEM of two independent experiments with five replicates. Representative photomicrographs are shown of untreated villous **(B)** or treated with rP21 **(C)**. Histological sections stained by Harris hematoxylin and eosin. Scale bar: 200 µm. In addition, the villi were pre-treated and/or infected with *T. cruzi* and subsequently with *T. gondii* **(D)** or infected with *T. gondii* and subsequently treated and/or infected with *T. cruzi* **(E)**. *T. gondii* intracellular proliferation was analyzed by means of the β-galactosidase assay and are presented as percentage (%) of *T. gondii* proliferation in relation to villi only infected with *T. gondii* (infected medium). Data are shown as means ± SEM of three independent experiments with eight replicates. Differences between groups were analyzed by means of one-way ANOVA test with Bonferroni’s multiple comparisons post-test. Significant differences in relation to villi only infected with *T. gondii* (^*^) or infected and treated with rP21 (^&^).

We also assessed the impact of rP21 and *T. cruzi* on *T. gondii* infection in human placental explants (**Figures 7A–E**).

Firstly, we observed that rP21 treatment did not cause cytotoxicity to villous explants (**Figure 7A**), as seen by the morphological analysis of the explants treated with rP21 similar to untreated villous (**Figures 7B, C**).

Next, *T. gondii* intracellular proliferation was evaluated in different situations. First, we observed that pre-treatment with rP21 decreased *T. gondii* proliferation in relation to villi only infected with *T. gondii* (infected medium) (^*^ *P* = 0.0236) (**Figure 7D**). However, pre-infection with *T. cruzi* in the absence or presence of rP21 increased *T. gondii* proliferation in relation to villi only infected with *T. gondii* (infected medium) (TCT: ^**^ *P* = 0.0011; TCT + rP21: ^**^ *P* = 0.0062), as well as in relation to the villi pre-treated with rP21 (^&&&&^ *P* < 0.0001) (**Figure 7D**).

We also observed that pre-infection with *T. gondii* and the subsequent treatment with rP21 and/or *T. cruzi* infection increased *T. gondii* proliferation compared to villi only infected with *T. gondii* (infected medium) (rP21: ^**^ *P* = 0.0075; TCT: ^*^ *P* = 0.021; TCT + rP21: ^*^ *P* = 0.0132) (**Figure 7E**).

### rP21 induces a decrease in TNF-α production, whereas *T. cruzi* infection increases IL-4 production and decreases IL-6, IL-8, MIF, and TNF-α

**Figure 8:**
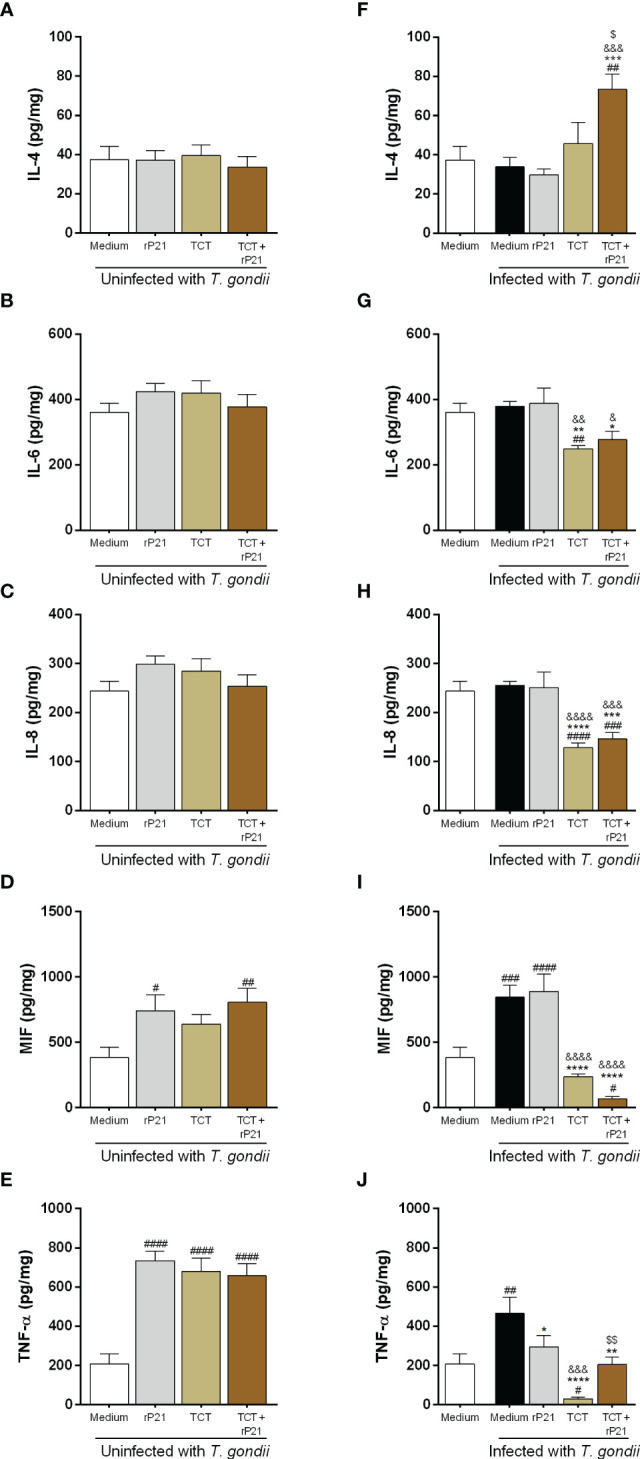
Cytokine production in human placental explants. The supernatants of villous infected or not with *T. gondii* and/or *T. cruzi* and treated or not with rP21 were submitted to ELISA assay for measurement of IL-4 **(A, F)**, IL-6 **(B, G)**, IL-8 **(C, H)**, MIF **(D, I)**, and TNF-α **(E, J)**. Data are presented in pg/mg according to the standard curve for each cytokine, and are shown as means ± SEM of three independent experiments with three replicates. Differences between groups were analyzed by means of one-way ANOVA test with Bonferroni’s multiple comparisons post-test or by means of Kruskall–Wallis test with Dunn’s multiple comparisons post-test. Significant differences in relation to uninfected and untreated villous (^#^), only infected with *T. gondii* (^*^) and treated with rP21 (^&^) or only infected with *T. gondii* (^*^), infected with *T. gondii* and treated with rP21 (^&^), or infected with *T. gondii* and *T. cruzi* (^$^).

Next, we measured cytokines in the supernatant of the villous explants infected with *T. gondii* and subsequently treated with rP21 and/or infected with *T. cruzi* (**Figures 8A–J**), as well as in the villous explants that were pre-treated with rP21 and subsequently infected with *T. gondii* (**Supplementary Figure 2**).

Regarding IL-4 production, no change was detected in the absence of *T. gondii* (**Figure 8A**). However, we observed that *T. gondii* infection and the subsequent infection and treatment with *T. cruzi–*rP21 increased the production of this cytokine in relation to uninfected and untreated villi (uninfected medium) (^##^ *P* = 0.0011), as well as in relation to villi only infected with *T. gondii* (infected medium) (^***^ *P* = 0.0004), infected and treated with rP21 (^&&&^ *P* = 0.0001), and infected with *T. cruzi* (^$^ *P* = 0.0101) (**Figure 8F**).

Concerning IL-6 production, no change was detected in the absence of *T. gondii* (**Figure 8B**). Nevertheless, we observed that infection with *T. gondii*/*T. cruzi* decreased IL-6 production in relation to uninfected and untreated villi (uninfected medium) (^##^ *P* = 0.0077) (**Figure 8G**). In addition, subsequent *T. cruzi* infection in the absence or presence of rP21 decreased the production of this cytokine in relation to villi only infected with *T. gondii* (infected medium) (TCT: ^**^ *P* = 0.0022; TCT+rP21: ^*^ *P* = 0.0178), as well as in relation to villi infected with *T. gondii* and treated with rP21 (TCT: ^&&^ *P* = 0.0011; TCT+rP21: ^&^ *P* = 0.0101) (**Figure 8G**).

Regarding IL-8 production, no change was verified in the absence of *T. gondii* (**Figure 8C**). However, we observed that *T. gondii*/*T. cruzi* infection in the absence or presence of rP21 decreased the production of this cytokine in relation to uninfected and untreated villi (TCT: ^####^ *P* < 0.0001; TCT+rP21: ^###^ *P* = 0.0009), as well as in relation to villi only infected with *T. gondii* (TCT: ^****^ *P* < 0.0001; TCT+rP21: ^***^ *P* = 0.0002) or infected and treated with rP21 (TCT: ^&&&&^ *P* < 0.0001; TCT+rP21: ^&&&^ *P* = 0.0004) (**Figure 8H**).

Concerning MIF production, we observed that villi only treated with rP21, or infected with *T. cruzi* and treated with rP21 increased the production of this cytokine in relation to uninfected and untreated villi (uninfected medium) (rP21: ^#^ *P* = 0.013; TCT+rP21: ^##^ *P* = 0.0039) (**Figure 8D**). In addition, villi only infected with *T. gondii*, or infected and treated with rP21, increased MIF production in relation to uninfected villi, whereas subsequent *T. cruzi* infection with rP21 decreased the production of this cytokine (Infected medium: ^###^ *P* = 0.0003; rP21: ^####^ *P* < 0.0001; TCT+rP21: ^#^ *P* = 0.0153) (**Figure 8I**). Furthermore, MIF production was lower in villi infected with *T. cruzi* in the absence or presence of rP21 in relation to villi only infected with *T. gondii* (^****^ *P* < 0.0001), as well as in relation to villi infected and treated with rP21 (^&&&&^ *P* < 0.0001) (**Figure 8I**).

Regarding TNF-α production, we observed that villi only treated with rP21, or infected with *T. cruzi* in the absence or presence of rP21 increased the production of this cytokine in relation to uninfected and untreated villi (uninfected medium) (^####^ *P* < 0.0001) (**Figure 8E**). Furthermore, villi infected only with *T. gondii* increased TNF-α production in relation to uninfected and untreated villi (uninfected medium), while subsequent *T. cruzi* infection decreased the production of this cytokine (infected medium: ^##^ *P* = 0.0035; TCT: ^#^ *P* = 0.0192) (**Figure 8J**). In contrast, villi infected with *T. gondii* and treated with rP21 or infected with *T. cruzi* in absence or presence of rP21 decreased TNF-α production in relation to villi only infected with *T. gondii* (infected medium) (rP21: ^*^ *P* = 0.0242; TCT: ^****^ *P* < 0.0001; TCT+rP21: ^**^ *P* = 0.0013) (**Figure 8J**). In addition, villi infected with *T. gondii*/*T. cruzi* decreased the production of this cytokine in relation to villi infected with *T. gondii* and treated with rP21 (^&&&^ *P* = 0.0001). However, villi infected with *T. gondii*/*T. cruzi* and treated with rP21 increased TNF-α production in relation to villi infected with *T. gondii*/*T. cruzi* (^$$^ *P* = 0.0088) (**Figure 8J**).

Furthermore, we observed that villi pre-treated with rP21 with subsequent infection with *T. gondii* did not alter IL-4 and IL-6 production in relation to villi infected or not with *T. gondii* (**Supplementary Figures 2A, B**). Nevertheless, pre-treatment decreased IL-8 production in relation to uninfected or infected villi (^##^ *P* = 0.002; ^***^ *P* = 0.0005) (**Supplementary Figure 2C**).

Concerning MIF production, we observed that villi infected with *T. gondii* increased the production of this cytokine in relation to uninfected villi (^##^ *P* = 0.0045), while pre-treatment with rP21 decreased MIF production in relation to villi infected (^*^ *P* = 0.023) (**Supplementary Figure 2D**).

Regarding TNF-α production, we observed that pre-treatment with rP21 decreased the production of this cytokine in relation to uninfected or infected villi (^#^ *P* = 0.0234; ^***^ *P* = 0.0003) (**Supplementary Figure 2E**).

### Proposed model for the action of rP21 and *T. cruzi* during *T. gondii* infection in human trophoblast cells and human villous explants

**Figure 9:**
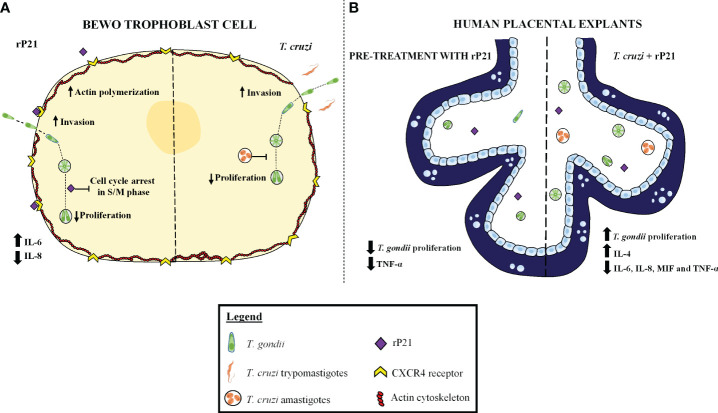
Proposed model of the role of rP21 and *T. cruzi* during *T. gondii* infection in human trophoblast cells and human villous explants. BeWo cells treated and/or infected with *T. cruzi* increase *T. gondii* invasion and decrease its proliferation. In addition, rP21 binds to CXCR4 and promotes actin polymerization, increasing *T. gondii* invasion, as well as arresting the *T. gondii* cell cycle in the S/M phase, upregulating IL-6, and downmodulating IL-8 production **(A)**. In human placental explants, the pre-treatment with rP21 decreases *T. gondii* proliferation and downmodulates TNF-α production, whereas post-treatment as well as *T. cruzi* infection increases *T. gondii* proliferation, in addition to the upregulation of IL-4 and downmodulation of IL-6, IL-8, MIF, and TNF-α **(B)**.

Based on our results, we propose a mechanism of action induced by rP21 and *T. cruzi* in *T. gondii* infection in *in vitro* and *ex vivo* models, as shown in **Figure 9**. In BeWo cells, we observed that *T. cruzi* infection and/or treatment with rP21 increase *T. gondii* invasion and decrease its proliferation. The greater invasion may be related to the binding of rP21 to CXCR4, leading to actin polymerization and favoring *T. gondii* invasion into the host cell. Conversely, the reduced *T. gondii* proliferation may be related to an arrest in the S/M phase of the parasite cell cycle without compromising its infectivity capacity, in addition to the upregulation of IL-6 and downmodulation of IL-8 (**Figure 9A**, left panel). In human placental explants, the pre-treatment with rP21 decreased *T. gondii* proliferation and downmodulated TNF-α production, whereas post-treatment and *T. cruzi* infection increased *T. gondii* proliferation, in addition to the upregulation of IL-4 and downmodulation of IL-6, IL-8, MIF, and TNF-α (**Figure 9B**, right panel). Thus, co-infection with *T. cruzi* may favor *T. gondii* transmission at the human maternal–fetal interface, and this phenomenon may be related to its protein P21.

## Discussion

In recent years, some studies have described coinfection situations involving bacteria, viruses, helminths, and protozoa in humans, which may lead to the aggravation of the diseases that they cause (68). Despite having few reports, coinfection by different protozoa can occur, especially in regions where there is a high incidence of infection by these parasites (69). The incidence of *Toxoplasma gondii* and *Trypanosoma cruzi* varies according to the region, with Latin America being one of the regions with elevated incidence of toxoplasmosis and Chagas disease. Both pathogens can be transmitted through the placenta, but there are still no reports in the literature that show infection by both protozoa in humans. In this sense, our work sought to assess whether infection by *T. cruzi* and one of its proteins, P21, could modulate *T. gondii* infection at the human maternal–fetal interface.

Initially, we analyzed whether the presence of *T. cruzi* could interfere in the *T. gondii* infection in different situations in BeWo cells, since an individual can be infected with *T. gondii* and later with *T. cruzi* or vice versa. Our data show that simultaneous infection with both parasites increased the invasive capacity of *T. gondii* in BeWo cells. However, pre-infection with *T. cruzi* did not alter *T. gondii* invasion. In contrast, pre- or post-*T. cruzi* infection decreased *T. gondii* proliferation in BeWo cells, suggesting that in situations of coinfection, the presence of *T. cruzi* can favor *T. gondii* entry in cells, but once inside the cell, its proliferation decreases. In agreement with our findings, *T. cruzi* infection has been shown to reduce HIV infection in human placentas and astrocytes, but the mechanisms involved are poorly understood (70, 71). Other studies show that *T. cruzi* parasitemia was increased in Swiss mice coinfected with *Schistosoma mansoni* (72). On the other hand, it has already been shown that infection by *T. cruzi* generates resistance in C57BL/6 mice to infection by *Plasmodium berghei* ANKA, conferring protection against severe malaria (73). Thus, it is possible to observe that coinfection situations are common and can modulate the course of infection of different pathogens. Although there are no reports in humans in the literature, coinfection by *T. gondii* and *T. cruzi* can happen, and this is the first study to evidence this phenomenon at the human maternal–fetal interface.

Since *T. cruzi* has a variety of soluble factors with biological properties that could be influencing this phenomenon, we sought to evaluate the effect of a protein secreted by the parasite, P21. Our results show that, regardless of the time treatment with rP21, the protein also increased the invasion and decreased the proliferation of *T. gondii*.

Subsequently, we evaluated the mechanisms triggered by rP21 that would be leading to this phenomenon. Da Silva et al. (41) demonstrated that this protein bound in a dose-dependent manner to HeLa cells and increased the invasion of *T. cruzi* extracellular amastigotes and trypomastigotes, but the mechanisms were unknown. Further studies demonstrated that rP21 binds to chemokine receptor CXCR4 and activates the PI3 kinase-signaling pathway, leading to actin polymerization and increasing phagocytosis of *Trypanosoma cruzi*, *Leishmania amazonensis*, and *T. gondii* in peritoneal macrophages (47). In this sense, we sought to assess whether CXCR4 as well as the actin cytoskeleton could be involved in the greater *T. gondii* invasion promoted by rP21 in our model. Our results showed that CXCR4 inhibition with AMD3100 reduced the *T. gondii* invasion promoted by rP21, confirming the action of this protein in the modulation of the *T. gondii* invasion. In addition, once rP21 binds to the receptor, it induced an increase in the actin cytoskeleton polymerization, favoring the entry of the parasite into the host cell. Although it is well known that the entry of *T. gondii* is an active process of the parasite (74, 75), the participation of the cell in this process is also important. Studies show that the activation of the PI3 kinase pathway by *T. gondii* is important for the invasion of the parasite in the host cell, and that this phenomenon correlates with the actin cytoskeleton polymerization, being an important mechanism for the entry of the parasite (76–78). Thus, the greater invasion promoted by rP21 can be explained by the activation of intracellular mechanisms that are important for the entry of *T. gondii*, being potentiated by the presence of the protein.

Next, we investigated what could be leading to a decrease in *T. gondii* proliferation promoted by rP21. In *T. cruzi* infection, this protein also decreases parasite proliferation as a mechanism to escape the host immune system (42, 43). In *T. cruzi* epimastigotes, the protein promoted a prolonged G1 phase and reduced the parasite protein synthesis (42). Here, through *T. gondii* cell cycle assays, we observed that rP21 promoted an increase in the S/M phase and decreased the G1 phase of the parasite, suggesting a retardation in *T. gondii* proliferation. Based on this result, we wondered whether the protein could be having a direct cytotoxic effect on the parasite, which could explain the decrease in proliferation. However, parasites that were treated with rP21 did not have their invasive capacity altered. These data suggest that, as observed previously for *T. cruzi*, rP21 may be inducing a decrease in *T. gondii* proliferation as a mechanism to protect the parasite from the host immune response. However, despite altering the *T. gondii* cell cycle, further investigations are needed to elucidate whether rP21 is acting directly on the parasite or whether this effect is species-specific.

P21 also has the capacity to induce an immune response through IL-4, IL-10, and IFN-γ production in *T. cruzi* infection, contributing to the control of parasite proliferation in the host organism and at the same time triggering the pathogenesis of Chagas disease (42, 43). We evaluated whether the decrease in *T. gondii* proliferation could be due to immune response induced by rP21 in BeWo cells, and we noticed that the protein increased IL-6 and decreased IL-8 production, while coinfection with *T. gondii–T. cruzi* in the presence of rP21 induced a slight decrease in MIF and increase in IL-6 production (not statistically significant).

IL-6 and IL-8 are multifunctional cytokines with pro-inflammatory properties and play different roles in *T. gondii* infection. Studies demonstrate that IL-6 is fundamental for controlling *T. gondii* infection in different models, such as human monocytes (79) and peritoneal macrophages (80), as well as at the maternal–fetal interface (52, 53, 61, 81). By contrast, IL-8 has been demonstrated as a crucial molecule for the activation of cellular mechanisms that help the spread of the parasite in the host organism (82, 83), and in BeWo trophoblastic cells, the *T. gondii* proliferation restriction was correlated with lower production of this cytokine (53). Therefore, the control of *T. gondii* proliferation promoted by rP21 in BeWo cells can be also explained by the increase in IL-6 and decrease in IL-8. It is plausible to suggest that the control of *T. gondii* proliferation by rP21 may be benefiting the establishment of the infection in the host cell, since the inflammatory response was not exacerbated, being important for the control of the infection but not completely eliminating the parasite, as well as for the host survival, since an exacerbated response could cause tissue damage and lead to its death, besides the fact that even in this pro-inflammatory environment, the viability of the parasite was not altered, as demonstrated in the reinfection assay. It is important to reinforce that our hypothesis is also verified by previous studies where the P21 also decreases *T. cruzi* proliferation as a mechanism to avoid the host immune response (42, 43).

Subsequently, we evaluated whether rP21 as well as *T. cruzi* would influence *T. gondii* infection in human placental explants from the third trimester of pregnancy, an *ex vivo* model of the human maternal–fetal interface. We observed that, as well as for cells, rP21 did not cause cytotoxicity to villous explants, maintaining the integrity of tissue morphology. In addition, pre-treatment with rP21 decreased *T. gondii* proliferation. Nevertheless, surprisingly, the treatment post-*T. gondii* infection and/or the presence of *T. cruzi* increased *T. gondii* proliferation in human placental explants.

It is known that *T. cruzi* infection promotes tissue disorganization in the placenta through the destruction of the syncytiotrophoblast, basal lamina, and type-I collagen in the villous stroma, compromising the integrity of the placental barrier (84–86). This disruption can facilitate the *T. gondii* entry and proliferation in the placenta and consequently reach the fetus. Since rP21 did not alter the viability or cause disorganization in the placental tissue, further studies are needed to assess which mechanisms the protein is triggering that benefit the growth of *T. gondii*.

In relation to cytokine production, our data show that *T. gondii* infection and post treatment with rP21 decreased TNF-α production, while subsequent infection with *T. cruzi* with or without rP21 increased IL-4 and decreased IL-6, IL-8, MIF, and TNF-α production, suggesting an anti-inflammatory profile. It is known that IL-4 is produced in acute *T. cruzi* infection to attenuate the exacerbated Th1 response (87). Although the Th1 response is important for the control of *T. gondii* infection, an uncontrolled inflammatory response can lead to tissue damage and host death, being necessary a contra-regulatory Th2 pattern. Even though a Th2 environment is important for a successful pregnancy, it may favor infection by other pathogens, such as *T. gondii*. Thus, we hypothesized that the increase in *T. gondii* proliferation in a situation of coinfection with *T. cruzi* is due to a decrease in pro-inflammatory cytokines and an increase in anti-inflammatory cytokines, while the decrease in TNF-α promoted by rP21 may also favor the parasite growth.

In addition, since pre-treatment with rP21 decreased *T. gondii* proliferation, we also evaluated the cytokine profile and observed a decrease in IL-8, MIF, and TNF-α, suggesting that controlling parasitism in this situation may not be involved with these cytokines. We hypothesize that the pre-treatment with rP21 may be triggering other intracellular mechanisms that resist infection, but at the same time, it may favor the parasite to remain in the tissue, yet further investigations are needed.

The differences in results between the models used in this study highlight the importance of using *in vitro* and *ex vivo* models for studies of the human maternal–fetal interface (88). It is clear that the use of cells alone is important to understand cell-specific intracellular mechanisms, but the use of the human placenta is necessary because it is a more representative model of the placental environment, since this model has not only the villous trophoblast, but also the extravillous trophoblast, as well as the syncytiotrophoblast, mesenchyme, and fetal blood vessels (89). Thus, the microenvironment of the placental explants is very different from a single isolate trophoblast. In this sense, it is plausible to declare that P21 is a protein able to benefit the establishment of *T. gondii* at the maternal–fetal interface, inducing the higher invasion and intracellular proliferation of this parasite.

In conclusion, we observed that *T. cruzi* infection may favor *Toxoplasma gondii* infection at the maternal–fetal interface. This can be partly explained by the P21 protein, since this protein helps the parasite enter the host cell through its binding to CXCR4 and actin polymerization. Once inside the cell, parasite proliferation decreases due to a parasite cell cycle arrest in the S/M phase, as well as the induction of a pro-inflammatory response, but which does not compromise the infective capacity of the parasite. In the placental tissue villous, coinfection with *T. cruzi* favors the *T. gondii* proliferation, while rP21 can decrease or increase its growth depending on the situation. This increase can be explained in part by the induction of an anti-inflammatory immune response, which may lead to greater congenital transmission of *T. gondii* from mother to fetus during pregnancy.

The results presented here bring forth new insights about the *T. gondii*–*T. cruzi* coinfection, since it is the first study to show a coinfection by both parasites, highlighting the importance of neonatal screening for both diseases, as well as in relation to P21, highlighting the importance of studies with this protein as a possible therapeutic target for the treatment of Chagas disease, since this protein, besides being important for the establishment of *T. cruzi* infection, may favor infection by other pathogens, such as *T. gondii*.

## Data availability statement

The original contributions presented in the study are included in the article/**Supplementary Material**. Further inquiries can be directed to the corresponding author.

## Ethics statement

The studies involving humans were approved by Ethics Committee of the Universidade Federal de Uberlândia, MG, Brazil, with approval number 3.679.426. The studies were conducted in accordance with the local legislation and institutional requirements. Written informed consent for participation in this study was provided by the participants’ legal guardians/next of kin.

## Author contributions

BB and GS designed the experiments. GS, ST, AF, RS, LL, JL, AR, NS, RO, MP, MB, RA, and BB performed the experiments. BB and GS analyzed the data. BB, AG, CS, and EF participated in the data analysis. BB and GS discussed the findings and reviewed the manuscript. All authors contributed to the article and approved the submitted version.

## Supplementary material

Supplementary Figure 1
*T. gondii* proliferation in BeWo cells. BeWo cells were infected with *T. gondii* and treated with rP21 and/or infected with *T. cruzi* simultaneously. *T. gondii* intracellular proliferation was analyzed with the β-galactosidase assay and presented as percentage (%) of *T. gondii* proliferation in relation to cells only infected with *T. gondii* (infected medium). Data are shown as means ± SEM of three independent experiments with eight replicates. Differences between groups were analyzed by one-way ANOVA test with Bonferroni’s multiple comparisons post-test. Significant differences in relation to cells only infected with *T. gondii* (^*^).

Supplementary Figure 2 Cytokine production in human placental explants. The supernatants of villous pre-treated with rP21 and infected with *T. gondii* were submitted to ELISA assay for measurement of IL-4 **(A)**, IL-6 **(B)**, IL-8 **(C)**, MIF **(D)**, and TNF-α **(E)**. Data are presented in pg/mg according to the standard curve for each cytokine, and are shown as means ± SEM of three independent experiments with three replicates. Differences between groups were analyzed by means of one-way ANOVA test with Bonferroni’s multiple comparisons post-test or by means of Kruskall–Wallis test with Dunn’s multiple comparisons post-test. Significant differences in relation to uninfected and untreated villous (^#^) or only infected with *T. gondii* (^*^).
